# Treatment with lamivudine versus lamivudine and thymosin alpha-1 for e antigen-positive chronic hepatitis B patients: a meta-analysis

**DOI:** 10.1186/1743-422X-6-63

**Published:** 2009-05-25

**Authors:** Yuan-Yuan Zhang, En-Qiang Chen, Jin Yang, Yu-Rong Duan, Hong Tang

**Affiliations:** 1 Center of Infectious Diseases, West China Hospital, Sichuan University, Chengdu 610041, Sichuan Province, PR China; 2 Division of Molecular Biology of Infectious Diseases, State Key Laboratory of Biotherapy, Sichuan University, Chengdu 610041, Sichuan Province, PR China; 3 The Chinese Cochrane Center/the Chinese Evidence- Based Medicine Center, West China Hospital, Sichuan University, Chengdu 610041, Sichuan Province, PR China

## Abstract

**Background:**

Currently, there is no evidence on the combination of lamivudine and thymosin alpha-1 on chronic hepatitis B patients. The aim of this study was to compare the effect of lamivudine monotherapy with that of lamivudine and thymosin alpha-1 combination therapy for the treatment of hepatitis B e antigen (HBeAg)-positive hepatitis B patients.

**Results:**

We searched PUBMED (from 1966 onwards), EMBASE (from 1966), CBMdisk (Chinese Biomedical Database, from 1978), CNKI (National Knowledge Infrastructure, from 1980), the Cochrane Central Register of Controlled Trials and the Cochrane Database of Systematic Reviews. Eight trials (583 patients in total) were identified. The lamivudine and thymosin alpha-1 combination treatment was significantly superior to lamivudine treatment in terms of ALT normalization rate (80.2% vs. 68.8%, P = 0.01), virological response rate (84.7% vs. 74.9%, P = 0.002), and HBeAg seroconversion rate (45.1% vs. 15.2%, P < 0.00001).

**Conclusion:**

Among HBeAg-positive patients, thymosin alpha-1 and lamivudine combination therapy may be more effective than lamivudine monotherapy, providing superior rates of biochemical response, virological response, and HBeAg seroconversion.

## Background

Hepatitis B is an infectious disease caused by hepatitis B virus (HBV) that affects more than 400 million people worldwide [1-4]. Chronic HBV infection is a serious problem associated with cirrhosis and hepatocellular carcinoma [5], which are becoming more prevalent worldwide, especially in Asia where the virus is often transmitted from mother to child at birth [6]. Chronic hepatitis B (CHB) infection is a dynamic state of interactions between the virus, host hepatocytes, and the host immune response. Immunological studies have found that impaired HBV-specific T cell reactivity is a major reason for the development of chronic infection. The HBV cytotoxic T lymphocyte response in patients with chronic HBV infection is generally weak or totally undetectable. The treatment for CHB consists of individualized, single-agent therapy with interferon or nucleoside analogues. Interferon, an immunomodulating agent, is effective in clearing the virus but is associated with adverse effects. Nucleoside analogues, such as lamivudine, can control HBV infection but have drug-resistant strains of HBV are increasingly prevalent [7]. They are effective in the therapy of chronic HBV infection but the efficacy is far from satisfactory. Persistent HBV infection represents a clear unmet need for improved antiviral therapeutic modalities.

Recently, some interesting data have recently emerged concerning the use of thymosin alpha-1 (Tα1) as monotherapy for CHB [8]. Tα1 is a 28-amino acid polypeptide produced synthetically but originally isolated from thymosin fraction 5, a bovine thymus extract containing a number of immunologically active peptides [9]. In vitro studies have shown that Tα1 can influence T-cell production and maturation and stimulate production of Th1 cytokines such as interferon-gamma and interleukin-2, and activate natural killer cell-mediated cytotoxicity [10,11]. It is an immunomodulatory agent that is able to augment some specific T lymphocyte functions, particularly ones that promote the T helper 1 cell responses involved in host antiviral defense [12].

Meta-analysis of 4 randomized controlled studies investigating the safety and efficacy of Tα1 monotherapy for the treatment of chronic hepatitis B showed that six months treatment of Tα1 (1.6 mg 2/week) almost doubled the sustained response rate compared with controls [13].

Monotherapy with lamivudine, interferon, or Tα1 is unlikely to be sufficient for the eradication of a CHB infection. Only a few randomized controlled clinical trials have been conducted to evaluate the efficacy of the combination of lamivudine and Tα1 in CHB. These trials were usually small, and their results were controversial. No meta-analysis on lamivudine versus lamivudine and Tα1 for treating CHB has been reported. Here we provide a comparison of lamivudine monotherapy to the combination of lamivudine and Tα1 in the treatment of hepatitis B e antigen (HBeAg)-positive patients.

## Results

### Studies identified

A total of 154 studies were identified by the searches. By scanning titles and abstracts, 105 redundant publications, reviews, case reports and meta-analyses were excluded. After referring to full texts, 41 studies that did not satisfy the inclusion criteria were removed from consideration. Eight studies were left for analysis which involved 583 patients, of whom 288 were included in monotherapy groups and 295 were included in combination therapy groups. Among them, 3 studies were published in English (Lee 2008 [14], Wu 2002[15], available by searching the database of PUBMED; Lin 2003[16], available by searching the database of The Cochrane Central Register of Controlled Trials); the others were published in Chinese, only by searching the database of CNKI [17-21]. We did not search citations in languages other than Chinese or English. Human trials were mostly in China because of the high prevalence of CHB in China, and thus results were mostly published in Chinese journals.

Table 1 shows the characteristics of the eight trials included in the meta-analysis. All had clearly stated inclusion and exclusion criteria. In addition, all studied populations with comparable baseline characteristics between the combination therapy and monotherapy groups, including age, sex, biochemical, and serological parameters. Four of the eight trials reported data for 12 months. All eight studies were randomized. Four studies mentioned withdrawal rates; however, none of the trials was blinded, and none mentioned the concealment of allocation clearly in the randomization process. Accordingly, we considered four studies as category B, and four as category C.

**Table 1:** Description of included randomized controlled trials

Author	Sample size (C/M)	Therapy period	Virological end-point	Follow-up period(m)	Study design	Grade
Lee 2008	62(31/31)	Tα1 24w+LAM 52w	HBV DNA(-)	0	RCT	B
Li 2005	68(34/34)	Tα1 52w+LAM 52w	HBV DNA(-)	0	RCT	C
Liang 2006	72(36/36)	Tα1 6m+LAM 12m	HBV DNA(-)	12	RCT	B
Lin 2003	72(35/37)	Tα1 26w+LAM 52w	HBV DNA(-)	12	RCT	B
Liu 2005	98(49/49)	Tα1 26w+LAM52w	HBV DNA(-)	0	RCT	C
Sun 2008	81(41/40)	Tα1 6m+LAM12m	HBV DNA(-)	12	RCT	C
Wu 2002	60(29/31)	Tα1 6m+LAM12m	HBV DNA(-)	12	RCT	B
Zhao 2007	68(33/35)	Tα1 6m+LAM12-18m	HBV DNA(-)	0	RCT	C

Abbreviations C: combination therapy; M: monotherapy; Tα1: thymosin alpha 1; LAM: lamivudine; w: weeks; m: months; RCT: randomized controlled trail.

### Biochemical response

**Figure 1:**
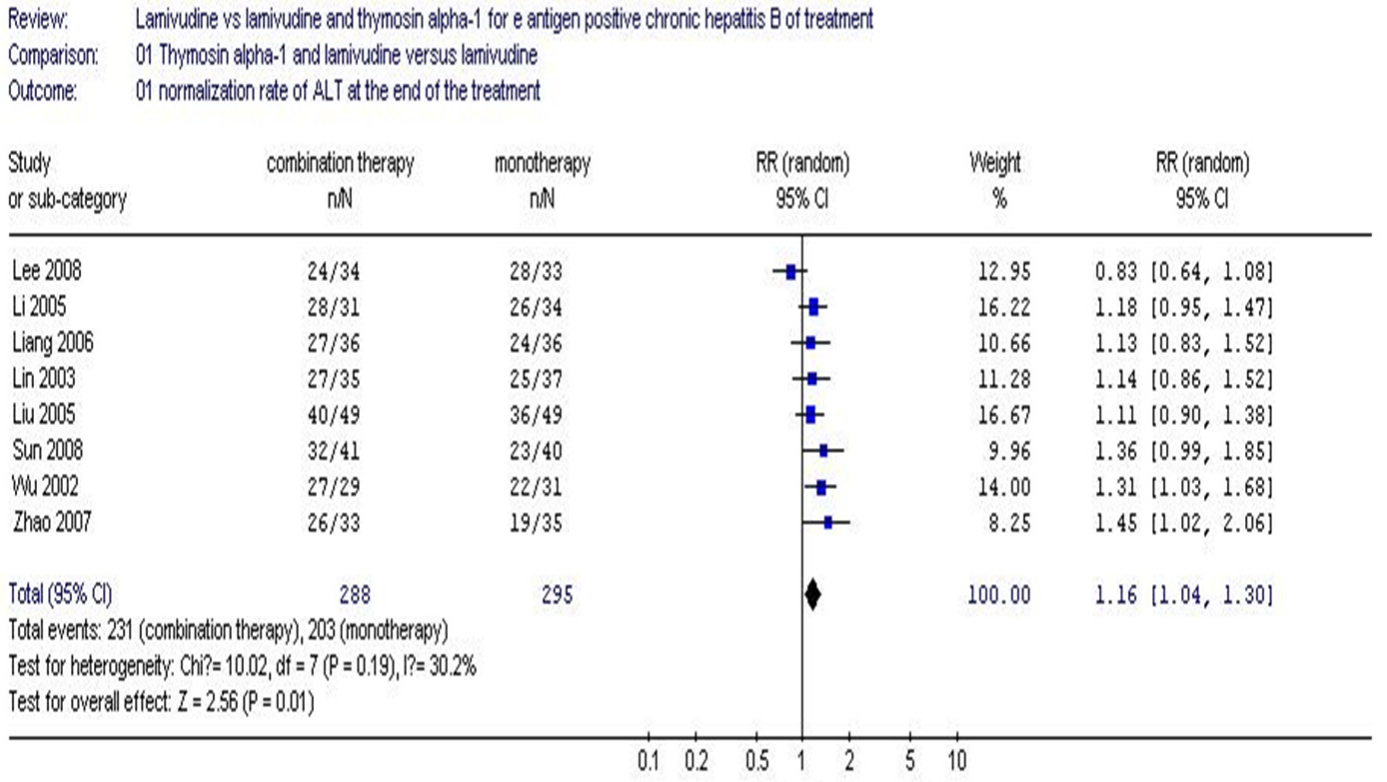
**Analysis of the normalization rate of ALT at the end of the treatment between lamivudine and thymosin versus lamivudine groups**.

The biochemical parameters at the end of the treatment are shown in Figure 1. The results of the eight studies showed normalization rates for ALT in the combination therapy group as 80.2%, compared to 68.8% in the monotherapy group at the end of treatment. No statistical heterogeneity was detected (χ^2 ^= 10.02, df = 7, *P *= 0.19, I^2 ^= 30.2%), allowing the use of a fixed effect model for meta-analysis. The difference of biochemical response rates at the end of treatment significantly favored the combination of Tα1 and lamivudine over lamivudine alone (RR 1.16, 95% CI 1.04–1.30, Z = 2.56, *P *= 0.01) (Figure 1).

**Figure 2:**
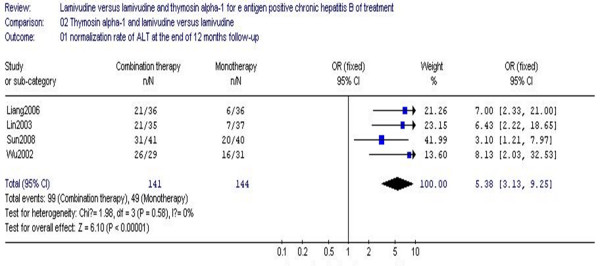
**Analysis of the normalization rate of ALT at the end of 12 months follow-up between lamivudine and thymosin versus lamivudine groups**.

The biochemical response of the patients in four studies at the end of 12 months' follow-up is shown in Figure 2. These included 285 patients and showed the biochemical response rates of ALT of the combination therapy group was 70.2%, compared to a 34.0% rate in the monotherapy group; no statistical heterogeneity (χ^2 ^= 1.98, df = 3, *P *= 0.58, I^2 ^= 0%) was found. The difference in biochemical response rates at the end of 12 months' follow-up reached statistical significance (RR 5.38, 95% CI 3.13–9.25, Z = 6.10, *P *< 0.00001) (Figure 2). Compared to lamivudine monotherapy, combination therapy with Tα1 and lamivudine was more effective in terms of biochemical response.

### Virological response

**Figure 3:**
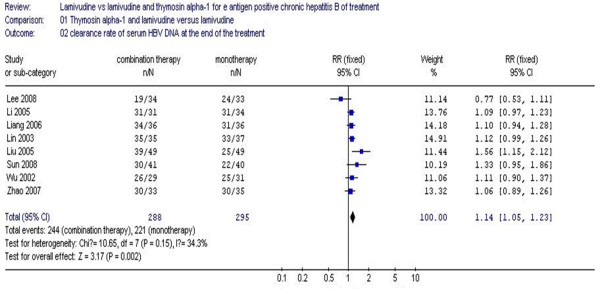
**Analysis of the virological response at the end of the treatment between lamivudine and thymosin versus lamivudine groups**.

The virological response at the end of the treatment is shown in Figure 3. The results of the eight studies showed the virological response rate of the combination therapy group was 84.7%, while the monotherapy group rate was 74.9%. There was no statistical heterogeneity (χ^2 ^= 10.65, df = 7, *P *= 0.15, I^2 ^= 34.3%), allowing use of the fixed effect model for meta-analysis. The difference of the virological response rates at the end of treatment between the two groups achieved statistical significance (RR 1.14, 95% CI 1.05–1.23, Z = 3.17, *P *= 0.002) (Figure 3).

**Figure 4:**
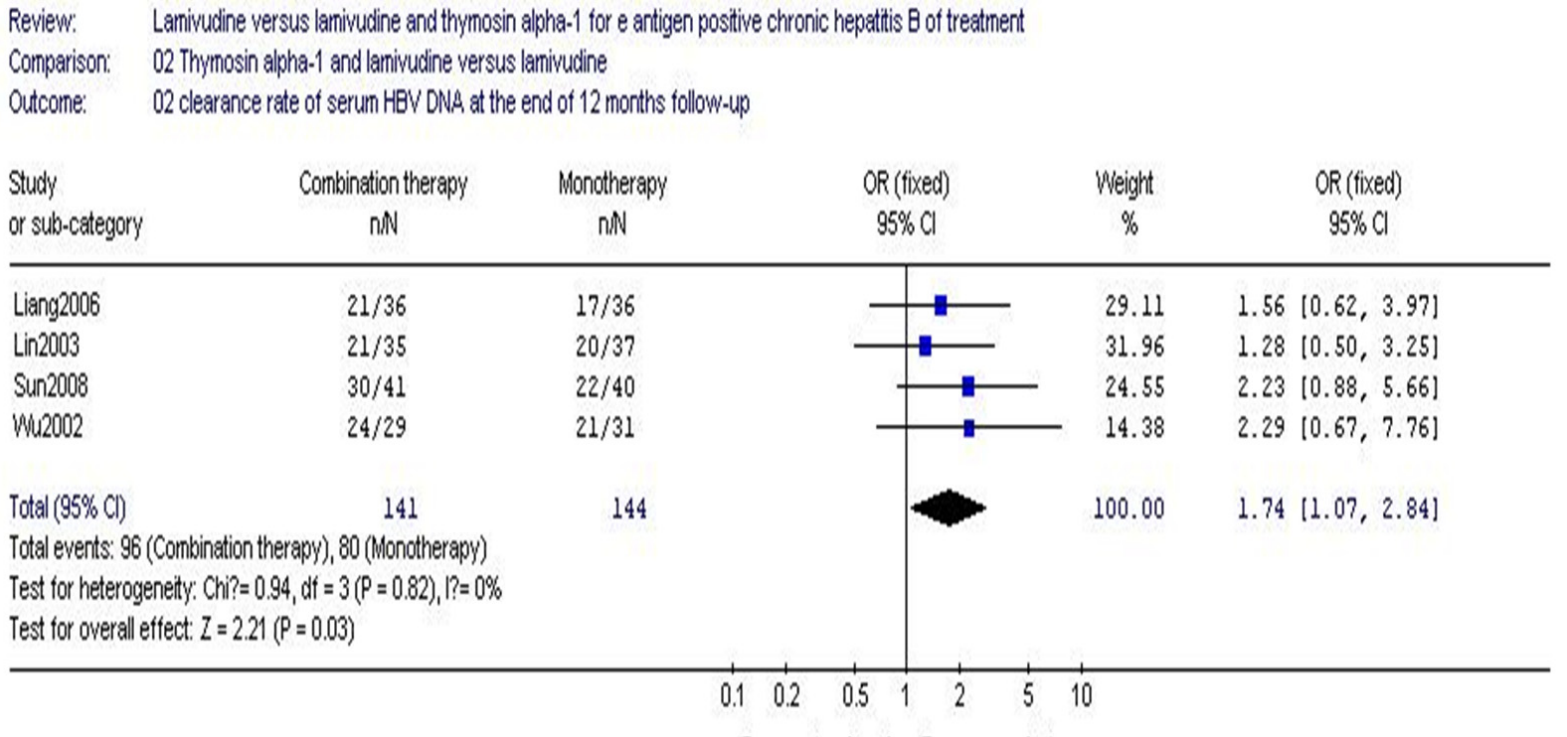
**Analysis of the virological response at the end of 12 months follow-up between lamivudine and thymosin versus lamivudine groups**.

The virological response at the end of 12 months follow-up is shown in Figure 4. The results of the four studies (285 patients) showed the virological response rate for the combination therapy group was 68.0%, while the monotherapy group response rate was 55.5% (Figure 4); no statistical heterogeneity was noted (χ^2 ^= 0.94, df = 3, *P *= 0.82, I^2 ^= 0%). The difference of virological response rates at the end of 12 months follow-up between the two groups was statistically significant (RR 1.74, 95% CI 1.07–2.84, Z = 2.21, *P *= 0.03) (Figure 4). When compared to lamivudine monotherapy, combination therapy with Tα1 and lamivudine was more effective as measured by virological response.

### Seroconversion of HBeAg to HBeAb

**Figure 5:**
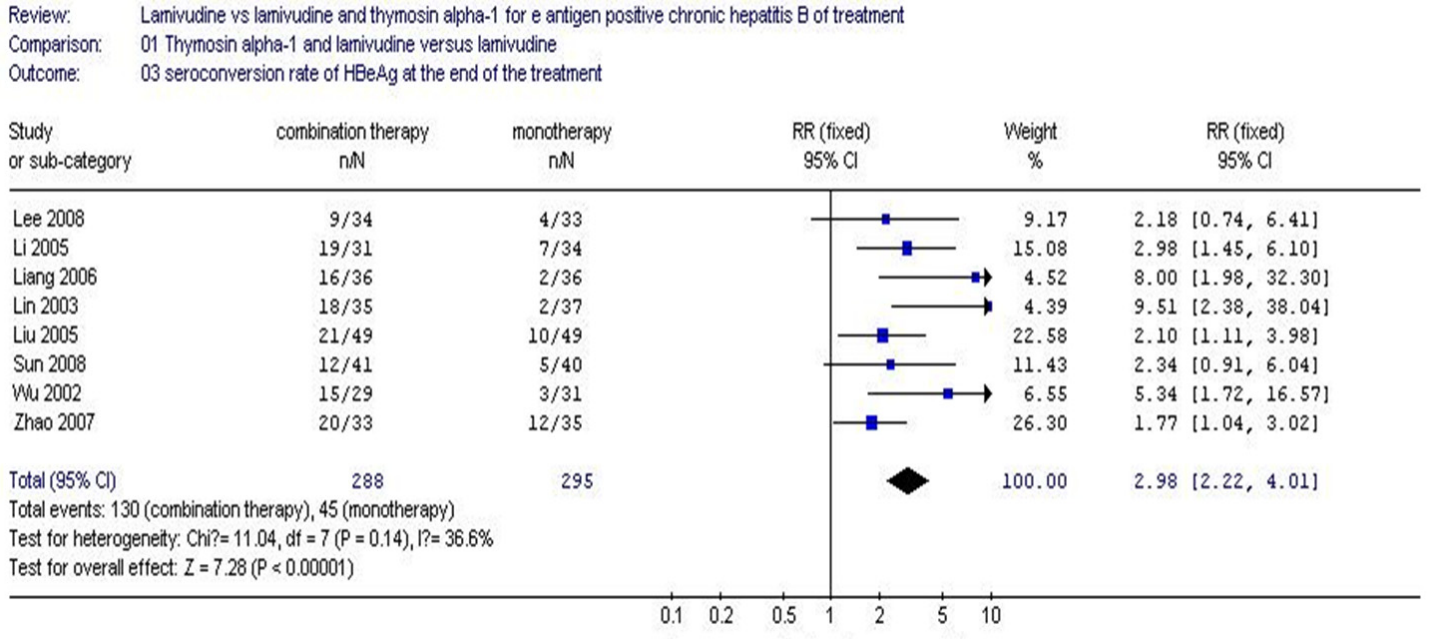
**Analysis of the HBeAg seroconversion rate at the end of the treatment between lamivudine and thymosin versus lamivudine groups**.

The seroconversion of HBeAg to HBeAb at the end of the treatment is shown in Figure 5. The seroconversion rate of patients receiving combination therapy was 45.1%, while the monotherapy group was 15.2% at the end of treatment. No statistical heterogeneity was found (χ^2 ^= 11.04, df = 7, *P *= 0.14, I^2 ^= 36.6%), allowing the use of a fixed effect model for meta-analysis. The difference of seroconversion rates at the end of treatment between the two groups achieved statistical significance (RR 2.98, 95% CI 2.22–4.01, Z = 7.28, *P *< 0.00001) (Figure 5). Therefore, in comparison to lamivudine monotherapy, combination therapy with Tα1 and lamivudine is more effective on seroconversion of HBeAg.

**Figure 6:**
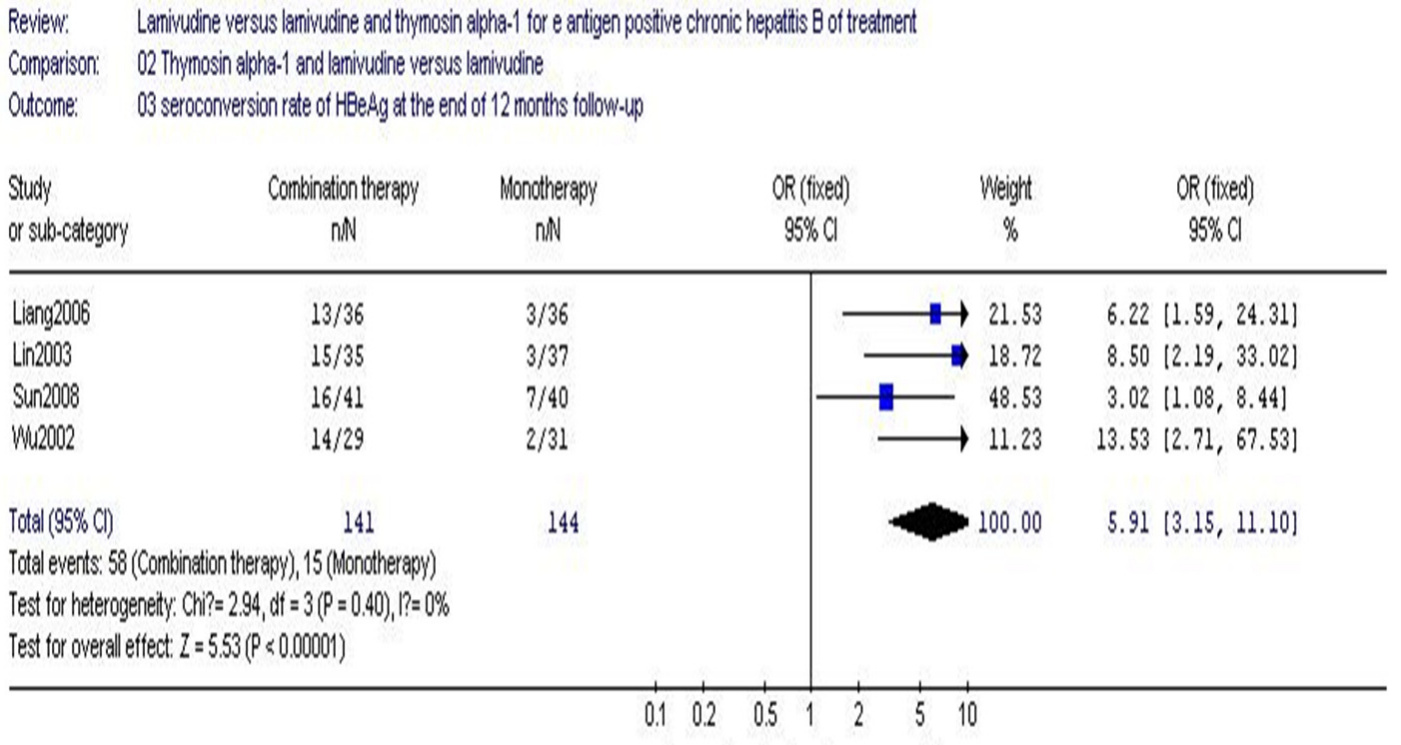
**Analysis of the HBeAg seroconversion rate at the end of 12 months follow-up between lamivudine and thymosin versus lamivudine groups**.

The seroconversion of HBeAg to HBeAb at the end of 12 months follow-up is shown in Figure 6. Four studies (which included 285 patients) showed the seroconversion rate of HBeAg for the combination therapy group as 41.1%, while the monotherapy group rate was 10.4% at the end of 12 months follow-up. No statistical heterogeneity (χ^2 ^= 2.94, df = 3, *P *= 0.40, I^2 ^= 0%). The difference in seroconversion rates at the end of treatment between the two groups achieved statistical significance (RR 5.91, 95% CI 3.15–11.10, Z = 5.53, *P *< 0.00001) (Figure 6). In comparison to lamivudine monotherapy, combination therapy with Tα1 and lamivudine was more effective with respect to seroconversion of HBeAg.

### Adverse events

No serious adverse events were reported in either group, and no biochemical abnormalities were reported in these studies. Patients reported nonspecific symptoms such as fatigue, mild dizziness, low fever, alopecia, and local discomfort at the injection site in the combination therapy group.

## Discussion

Meta-analysis is a statistical technique for assembling the results of several independently conducted but closely related studies to arrive at a single numerical estimate of risk or benefit. The suboptimal outcomes of current hepatitis B monotherapies have prompted the notion of combination therapy to achieve a synergistic effect [2]. In the present study, we considered HBeAg-positive patients, who tend to have more active disease and are at higher risk for complications. We evaluated combination therapy of Tα1 and lamivudine for CHB patients, pooling data from all pertinent randomized-controlled trials. If successful, this meta-analysis will help reach evidence-based conclusions, resolve the controversy surrounding this topic, and direct further investigation.

In this analysis, it appears that the combination of Tα1 (1.6 mg subcutaneously, twice a week) for a minimum of 24 weeks and lamivudine (100 mg orally, daily) for a minimum of 52 weeks was more effective than lamivudine monotherapy at the end of treatment. The combination therapy provided superior rates of sustained virological response (*P *= 0.01), biochemical response (*P *= 0.002), and HBeAg seroconversion (*P *< 0.00001) than did monotherapy. Further, combination therapy may improve the rates of ALT normalization (*P *< 0.00001), HBV DNA loss (*P *= 0.03), and HBeAg seroconversion (*P *< 0.00001) at the end of 12 months' follow-up.

In general, the objectivity and accuracy of meta-analysis rely on the availability of high-quality studies. We should consider results of the current analysis cautiously for several reasons. Firstly, blinding of subjects and clinicians was difficult because the combination therapy group received injected Tα1 and orally administered lamivudine while the monotherapy group only received orally administered lamivudine. As a result, none of the studies included in the analysis was double-blinded; however, it is unlikely that the lack of blinding could affect the outcomes assessed. In such a study design, blinding could be achieved only if both the combination therapy group and monotherapy group received oral and injected trial medications (i.e., the monotherapy group could receive placebo injection). Secondly, none of the trials described the method used to generate the allocation sequence. Despite these potential sources of bias, randomization was adequate in the eight trials as shown by the baseline equivalency of experimental groups. Finally, HBV DNA was measured using a hybridization assay in one trial (Lee, 2008), and HBV DNA was measured by polymerase chain reaction in the other trials. The different HBV DNA assays used in the different trials may also have caused additional variability in the sensitivity of HBV DNA detection and thus in the estimate of efficacy. Additional issues include publication bias, small trial sizes, and a high rate of studies that were conducted in China. In other countries, the efficacy and safety of Tα1 and lamivudine versus lamivudine for treating CHB have not been largely explored, potentially resulting in language bias.

## Conclusion

In summary, thymosin alpha-1 and lamivudine combination therapy may be more effective than lamivudine monotherapy among HBeAg-positive patients, providing superior rates of biochemical response, virological response, and HBeAg seroconversion. And more high-quality, well-designed, randomized controlled trials that are adequately powered are clearly needed to guide evolving standards of care for CHB. Randomization procedures should be clearly described, allocation concealment should be emphasized, and the approaches should be reported. Blinding should be conducted, though this may be difficult.

## Methods

### Inclusion criteria

For inclusion in our analysis, studies were required to meet several criteria. First, the study population must be 18–75 years of age and diagnosed with HBeAg-positive CHB, with HBV DNA positivity lasting for at least 6 months, and must show elevated alanine transaminase (ALT) levels. Gender and ethnic origin were not considered. Second, trials must have been described as randomized Third, the intervention(s) must have included lamivudine monotherapy and combination therapy with lamivudine and Tα1. Monotherapy with lamivudine (100 mg orally, daily) must have been for at least of 52 weeks, and combination therapy must have been with lamivudine (100 mg orally, daily) for at least 52 weeks and Tα1 (1.6 mg subcutaneously, twice a week) for at least 24 weeks. Fourth, published data must include biochemical and virological response rates, seroconversion rates (HBeAg to HBeAb), and adverse effects.

### Exclusion criteria

Trials were excluded if they did not meet the inclusion criteria above. Animal or in vitro studies were also excluded, as were review articles, duplicate or redundant publications, and letters to the editor. Studies involving patients with antibodies to human immunodeficiency virus (HIV), hepatitis C virus (HCV), hepatitis D virus (HDV) or hepatitis E virus (HEV), studies of patients with decompensated liver disease, evidence of other forms of liver disease, or a history of malignancy were also excluded.

### Search strategy

Retrieval of trials published up to September, 2008 was performed through PUBMED (from 1966 onwards), EMBASE (from 1966), CBMdisk (Chinese Biomedical Database, from 1978), and CNKI (National Knowledge Infrastructure, from 1980). The Cochrane Central Register of Controlled Trials and the Cochrane Database of Systematic Reviews were also searched. The search process was designed to find initially all trials involving terms: "Hepatitis B", "e antigen positive", "thymalfasin", "thymosin alpha-1" "lamivudine", "randomized controlled trial", "randomization", "controlled study", "multicenter study", "double blind procedure", "single blind procedure" (and multiple synonyms for each term). Computer searches were supplemented with a manual search. Search results were downloaded to a reference database and further screened.

### Definition of main outcomes

Published data at the start and the end of the therapy include the efficacy measure, i.e. biochemical and virological response rates, seroconversion rates (HBeAg to HBeAb), and adverse effects. Biochemical response was defined as normalization of ALT levels. Virological response was defined as attainment of undetectable (or below 1000 copies/mL) levels of HBV DNA, as determined by polymerase chain reaction or measured using a hybridization assay. The serum HBV markers were detected by the Enzyme-Linked Immunosorbent Assay. We analyzed the outcomes at the end of the active treatment phase.

### Methods of review

#### Data extraction

Two reviewers independently selected the trials and performed the data extraction. Discrepancies were resolved by discussion among reviewers. In some cases, original principal investigators were contacted to collect information that was collected but not published.

#### Quality assessment

The overall quality of each study was assessed in accordance with the Cochrane format [22], using a grading scheme for each of four main aspects, each classified into three grades (A, B, and C) as follows: 1) quality of randomization, 2) quality of allocation concealment, 3) quality of blinding, and 4) quality of the description of withdrawals and dropouts. The grades were: A) adequate, with correct procedures, B) unclear, without a description of methods, and C) inadequate procedures, methods, or information. Based on these four criteria, the studies could be divided into three groups. "A" studies had a low risk of bias for studies and were scored with A grades for all items; "B" studies had a moderate risk of bias for studies with one or more B grades; "C" studies had a high risk of bias and were those with one or more C grades.

### Statistical methods

Statistical analysis was carried out using Review Manager (version 4.2) provided by The Cochrane Collaboration. Dichotomous data were presented as relative risk (RR) and continuous outcomes as weighted mean difference (WMD), both with 95% confidence intervals (CI). The overall effect was tested using Z scores, with significance being set at *P *< 0.05. Meta-analysis was performed using fixed-effect or random-effect methods, depending on absence or presence of significant heterogeneity [23]. Statistical heterogeneity between trials was evaluated by the chi-squared and I square (I^2^) tests, with significance being set at *P *< 0.10. In the absence of statistically significant heterogeneity, the fixed-effect method was used to combine the results. When heterogeneity was confirmed (*P *= 0.10), the random-effect method was used.

## Competing interests

The funding source had no influence on study design, in the collection, analysis, and interpretation of the data, in the writing of the manuscript, or in the decision to submit the manuscript for publication. The contents are solely the responsibility of the authors and do not necessarily represent the views of the funding source.

## Authors' contributions

HT conceived the study, provided fund supporting and revised the manuscript critically for important intellectual content. YZ made substantial contributions to its design, acquisition, analysis and interpretation of data. EC, JY and YD participated in the design, acquisition, analysis and interpretation of data. All authors approved the final manuscript.
